# Occurrence of enterotoxigenic *Escherichia coli* in the aquatic environment and impact of climatic factors

**DOI:** 10.3389/fenvs.2025.1593899

**Published:** 2025-07-15

**Authors:** Fatema-Tuz Johura, Marzia Sultana, Jade Lewis, Abdus Sadique, David A. Sack, Richard B. Sack, Munirul Alam, Subhra Chakraborty

**Affiliations:** 1 Department of International Health, Johns Hopkins Bloomberg School of Public Health, Baltimore, MD, United States,; 2 Infectious Diseases Division, icddr,b Formerly Known as International Center for Diarrheal Disease Research, Dhaka, Bangladesh

**Keywords:** enterotoxigenic *E. coli*, environment, climate, ecological parameters, Bangladesh

## Abstract

**Background:**

The prevalence of ETEC in the environment could be influenced by environmental factors, which may contribute to seasonal outbreaks. This study assessed the seasonal prevalence of the ETEC population in water bodies in relation to ecological changes in Bangladesh.

**Method:**

The presence of ETEC in water, plankton, and sediment samples collected from water bodies in Mathbaria, a southern coastal region, was investigated using quantitative PCR (qPCR) and an extensive culture method over a year. Concurrently, the physico-chemical parameters of the water were measured. We also tested aquatic samples from Chhatak, a northern hilly region.

**Results:**

ETEC was detected in a high proportion (56%–78%) in water, plankton, and sediment samples from both sites. In Mathbaria, higher abundance of ETEC was noted in fall and spring, followed by summer and lowest in the monsoon. ETEC positivity was high in winter by qPCR, but no viable ETEC could be isolated. Environmental conditions such as neutral to low alkaline pH, elevated dissolved oxygen levels, and warmer water temperatures showed a trend of association with increased ETEC prevalence.

**Conclusion:**

The persistence of ETEC in environmental water and their association with ecologic factors may help to explain seasonal illnesses in people.

## 1 Introduction

Enterotoxigenic *Escherichia coli* (ETEC) is a primary bacterial source of diarrhea among children under 5 in low- and middle-income countries, and in older people and it is a significant contributor to traveler’s diarrhea (World Health Organization, 2006; Fischer Walker et al., 2010 Crossman et al., 2010; Kotloff et al., 2013; Lamberti et al., 2014; Chakraborty et al., 2024). It is estimated that ETEC causes about 220 million diarrhea episodes globally, with about 75 million episodes in children under 5 years of age. ETEC is often the first bacterial pathogen that causes diarrhea in neonates and children and is responsible for two to five diarrheal episodes during the first 3 years of life (Khalil et al., 2018; Kotloff et al., 2013).

In Bangladesh, infections caused by ETEC follow a typical biannual seasonality with two seasonal peaks, a larger peak at the beginning of the summer, that is, the spring (April to June), and a smaller peak in the fall (September to November), after the monsoons, although the infections are endemic throughout the year (Qadri et al., 2005; Chakraborty et al., 2024). These biannual seasonal picks might be driven by the changes in the environmental parameters.

Like ETEC, *Vibrio cholerae* also follows two seasonal peaks in Bangladesh (Alam et al., 2006b). These seasonal peaks are shown to be linked with changes in the population of *V. cholerae* in the aquatic bodies like ponds and rivers, which are used by the locals for their daily activities (Alam et al., 2006b). The population of *vibrio* in the water is impacted by the changes in the aquatic environmental parameters (Huq et al., 2005). The analysis of environmental sampling and the environmental and clinical data has shown strong connections between the presence of *V. cholerae* and factors such as water temperature, water depth, rainfall, conductivity, and copepod count (Huq et al., 2005). Fluctuations in environmental factors like temperature and salinity, alongside biological elements like changes in plankton populations, are directly linked to the occurrence and spread of cholera over time (Huq et al., 2005). Plankton serves as a reservoir of *V. cholerae* in the aquatic environment. During the summer, phytoplankton blooms occur in Bangladesh, followed by zooplankton blooms in September and October. Cholera outbreaks almost always follow the zooplankton bloom (Huq et al., 1995).

Since ETEC follows a similar seasonal pattern as *V. cholerae*, it is possible that ETEC can survive in the aquatic bodies and their population could be influenced by the seasonal environmental parameters. A few studies have demonstrated the presence of ETEC in surface water (Begum et al., 2005; Begum et al., 2007; Akter et al., 2013). However, it is not known if their presence, abundance, persistence, and survival in aquatic ecosystems are influenced by environmental parameters which may contribute to the seasonal outbreaks of ETEC diarrheal disease. Additionally, the role of plankton as a potential reservoir for ETEC has not been studied. Addressing these questions would help design strategies to improve water quality management and public health strategies aimed at controlling waterborne ETEC infections and outbreaks.

In this study, we examined how the prevalence of ETEC in surface water, plankton, and sediments changes throughout the seasons in the rural coastal area of southern Bangladesh. Both quantitative PCR and an extensive culture method were employed to detect ETEC in the environmental samples. Additionally, we investigated the impact of variations in aquatic environmental parameters (such as air and water temperature, water conductivity, salinity, dissolved oxygen, pH, turbidity, and total dissolved solids) on the occurrence of ETEC in aquatic bodies. To explore potential geographical variations in the occurrence of ETEC in water bodies, we also analyzed samples from the hilly area of Chhatak in northern Bangladesh. Understanding the influence of climate on the ETEC population in the aquatic bodies used for daily household activities in Bangladesh will provide insights into transmission patterns and seasonal outbreak mechanisms.

## 2 Materials and methods

**FIGURE 1:**
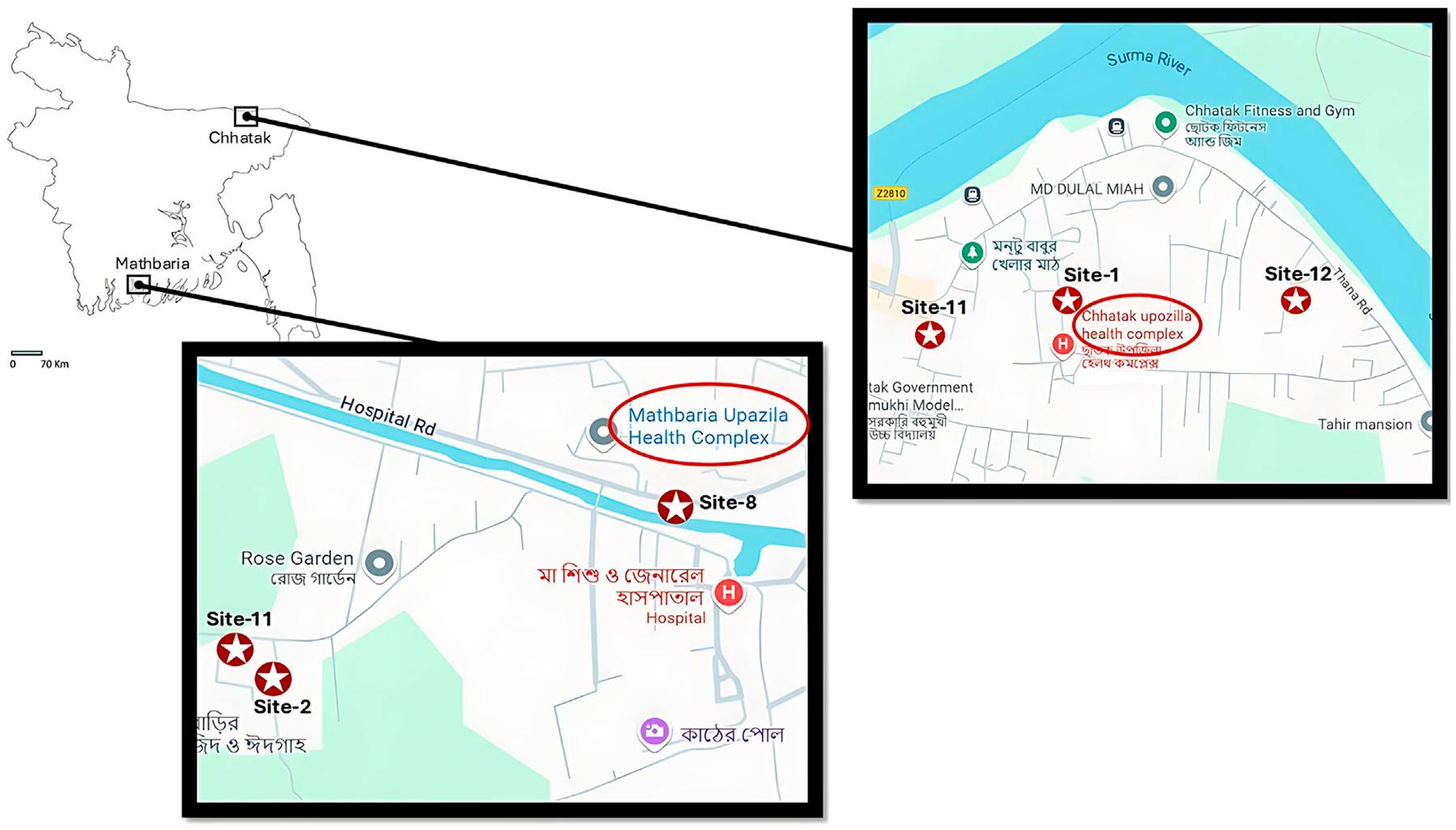
Two locations, Mathbaria and Chhatak, served as sites for the collection of water, plankton, and sediment samples. Mathbaria is situated in the southern coastal region, while Chhatak is in the northeastern hilly part of Bangladesh. The larger sections of the map illustrate 3 different sampling sites in Mathbaria and Chhatak. Mathbaria is a coastal village located adjacent to the Bay of Bengal, southern part of Bangladesh. The red circle with star shows the sampling sites: Site-2 (pond), Site-8, (Mathbaria Canal) and Site-11 (pond). The positions on the map were determined by Global Positioning System (GPS). The red circle shows the Mathbaria Upazila health complex. Chhatak is situated in the flood plains of the river Brahmaputra, northeastern part of Bangladesh. The red circle with star shows the sampling sites: Site-1, Site-11, and Site-12. The positions on the map were determined by Global Positioning System (GPS). The red circle shows the Upazila health complex.

This study was conducted in two geographically distinct locations, Chhatak in the north and Mathbaria in the southern coast of Bangladesh in 2015 (Figure 1). Each sampling location included three sites (two ponds, one canal in Mathbaria, and three ponds in Chhatak). Sampling sites were carefully selected, with attention to terrain and human population encompassed and availability of hospital service. In selecting sites for sample collection, attention was given to whether neighboring villagers used the water bodies and sought care for diarrhea at the study hospital, a primary healthcare facility, Thana Health Complex (THC) (Chakraborty et al 2024). The clinical and environmental sampling were conducted simultaneously. The clinical sampling and the results were reported previously (Chakraborty et al 2024; Johura et al 2024).

Environmental sampling in Mathbaria was conducted to determine the physicochemical parameters of the natural bodies of water used by villagers as a source of drinking and household activities and their relationship to the prevalence of ETEC. Environmental samples from Chhatak were used to determine the prevalence of ETEC bacteria in water bodies.

### 2.1 Description of study areas

- Mathbaria: Mathbaria is located adjacent to the Bay of Bengal, approximately 400 km southwest of Dhaka (Capital city of Bangladesh). Mathbaria is an administrative unit under the district of Pirojpur, with a police station and a THC. The major river, Baleshwar, flows along the western boundary of Mathbaria, on the other side of which is located a tropical mangrove forest of the Sundarbans, the temporary island system of that part of the Bay of Bengal. In this study, samples from Mathbaria were collected biweekly during the diarrhea “high” seasons in March-May and September-November and monthly during diarrhea “low” seasons in December and June-August. The “high” and “low” diarrhea seasons were determined based on patterns from previous years. This strategy was adopted with more sampling days during the high seasons, to allow for an increased number of cases to be detected and documented. Among the three study sites included in Mathbaria are two man-made ponds (site 2 and 11) and a canal (site 8) (Figure 1)
- Chhatak: Chhatak is in the Sunamganj district, 40 km from the divisional town of Sylhet. The Surma River flows through the district and is heavily polluted with industrial wastes from an adjacent pulp mill and a cement factory. During monsoon flooding, the Surma River overflows its banks. In this study, samples from Chhatak were collected monthly during March-May. Among the three study sites included in this district are three ponds (site 1, site 11 and site 12) (Figure 1)

### 2.2 Collection of environmental samples

Water, plankton, and sediment samples were collected between March and December 2015 from Mathbaria and between March and May 2015 from Chhatak and processed as previously reported (Alam et al., 2006b). For the collection of plankton samples, 100 L of water was filtered successively through 64 μm and 20 μm mesh nylon nets (Millipore Corp., Bedford, MA) (placing the 64 μm net sequentially in front of the 20 μm nylon net, with each having a collection bucket at the base), and 50 mL of the concentrates were collected in sterile glass bottles initially as a crude measure of plankton. Also, during the process of filtration, 500 mL of filtrate from the 20 μm net was collected using sterile dark Nalgene bottles (Nalgene Nunc International, St. Louis, Mo.) and analyzed for bacteria in the water samples. Sediment samples were collected using a core sampler (constructed at icddr,b) and transported to the laboratory in 125 mL sterile glass bottles (Huq et al., 2005). All samples were collected with an aseptic technique and transported to the lab at icddr,b, Dhaka, Bangladesh, and processed within 12–16 h of collection (Alam et al., 2006a).

### 2.3 Physiochemical parameters

Air and water temperature, water conductivity, and salinity were measured using a portable meter (HACH model CO150 conductivity meter). Dissolved oxygen and pH were also measured, using a portable HACH (model DO175) dissolved oxygen meter and Orion field pH meter (model 210A, Orion Laboratories), respectively. Water turbidity was determined with a HACH 2100Q turbidimeter, while total dissolved solids were measured using a portable HACH MP-4 meter.

### 2.4 Microbiological analysis

#### 2.4.1 Processing of samples

After receiving all the samples at icddr,b laboratory, water samples were concentrated by filtering through a 0.22 μm bacteriological membrane filter (MilliporeCorp., Bedford, MA), and the retained content on the membrane filter was washed into phosphate-buffered saline (pH 8.0) which was used for qPCR and culture, as described below (Alam et al., 2006b). For each plankton sample, 80 mL was taken and further concentrated to 10 mL by filtering through a 20 μm mesh nylon filter and homogenizing in a Teflon-tipped tissue grinder (Wheaton Scientific, Millville, NJ) using a Stead Fast stirrer (model 300, Fisher Scientific) (Huq et al., 2005). Ten grams of sediment were vortexed in 90 mL sterile physiological saline for 2 min (Huq et al., 2005).

#### 2.4.2 Detection of virulence genes

qPCR: Genomic DNA was extracted directly from the concentrated water, plankton, and sediment samples by using *Qiagen* DNeasy^®^ Blood & Tissue kit (Qiagen, Hilden, Germany) as per manufacturer instruction. The SYBR green real-time PCR assays (single plex reaction) were used for the detection of enterotoxigenic *E. coli* (ETEC) specific genes *lt*, *sth*, and *stp* (Connor et al., 2022) using the extracted DNA from the aquatic samples. The reaction mix contained (20 μL) 2X Fast SYBR green master mix (Applied Biosystems) with primers and DNA template. After amplification, a melting-curve analysis of the amplified DNA was performed using the default temperature profile. ETEC strain H10407 (O78:H11, *lt*^+^ *sth*^+^ *stp*^+^) was used as positive control.

#### 2.4.3 Enrichment and culture

Following processing, all the water, plankton, and sediment samples were enriched in MacConkey broth at 37°C for 18–24 h before being plated. Approximately 100 μL of enriched broth was serially diluted and spread onto a MacConkey agar plate using a sterile spreader, then incubated at 37°C for 16–18 h. From each sample, 50 isolated lactose-fermenting *E. coli*-like colonies were chosen for testing ETEC-specific genes *lt*, *sth* and *stp* using PCR (Chakraborty et al., 2024).

#### 2.4.4 Conventional PCR

DNA was extracted from the *E. coli* isolates by the boiling method. Specific primers to detect ETEC-specific genes *lt*, *sth*, and *stp* genes were used for PCR (Chakraborty et al., 2024).

### 2.5 Data analysis

To investigate the differences in the mean of the measurements of the environmental parameters between ETEC-positive and ETEC-negative samples, Welch’s two-sample t-tests were conducted. Univariate logistic regression models explored the relationship between individual environmental factors and ETEC status across various sample types. Pearson’s correlation analyses were subsequently performed to examine potential associations between ETEC status and environmental parameters within the three sample types. For water temperature, the readings from two probes placed in different areas of each water body were averaged to ensure an accurate analysis of its relationship with ETEC status. Similarly, dissolved oxygen (DO) values were averaged from three different measurements (measurements were taken from different areas of the water bodies at each collection time). All statistical analyses were performed using R (version 4.3.1, 2023–06-16) and can be found in the Supplementary Material. In the manuscript, ETEC with only LT toxin gene is designated as LT-ETEC; ETEC with only ST toxin gene as ST-ETEC and ETEC with both the LT and ST toxin genes as LT + ST-ETEC.

## 3 Results

A total of 187 water, plankton, and sediment samples were collected from Mathbaria and Chhatak during the study. The percentage of samples testing positive for ETEC by qPCR is shown in Table 1.

Using qPCR for ETEC toxin genes, 68.42% of the surface water was positive in the coastal area of Bangladesh. ETEC was equally abundant in plankton (67.92%) and sediment (70%) samples in this area. Similarly, in Chhatak, the water (77.78%), plankton (77.78%), and sediment (55.55%) samples were heavily contaminated with ETEC. In Mathbaria, the occurrence of ST-ETEC was more prevalent across all three sample types, followed by LT + ST-ETEC, while very few LT-ETEC positive samples were noted. In contrast, in Chhatak, the frequency of LT + ST-ETEC was higher in all three sample types, relative to LT-ETEC and ST-ETEC (Table 1).

Since samples were only collected for 3 months March to May, we could not perform a seasonal analysis of the Chhatak samples as we did with those from Mathbaria. Nevertheless, the presence of ETEC in water, plankton, and sediment in Chhatak was comparable to that in Mathbaria.

We found a stronger and consistent relationship between the seasonality and the presence of ETEC for site 8 in Mathbaria, although the general trends were similar throughout all the sites. The data from site eight is presented in this report as a representative of the water bodies.

### 3.1 Seasonal occurrence of ETEC in water, plankton and sediment

**FIGURE 2:**
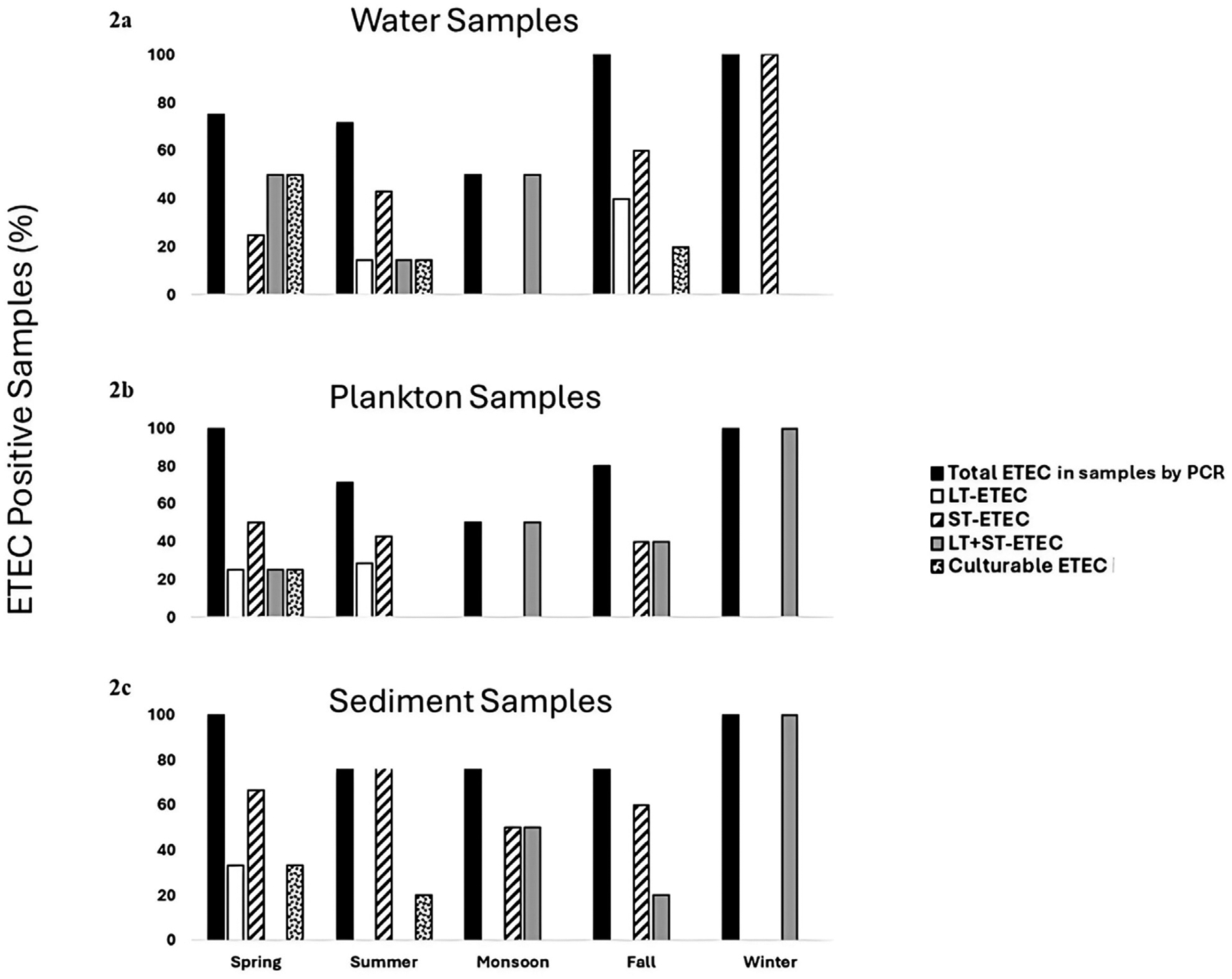
Percentages of samples positive for total ETEC, LT-ETEC, ST-ETEC, and LT + ST-ETEC determined by qPCR and culture (culturable ETEC) **(a)** water **(b)** plankton and **(c)** sediment in Mathbaria.

The positivity rates of total ETEC in water was similar in the spring (75%) and summer (71.43%), while reduced in monsoon to 50% (Figure 2a). Again, a sharp increase in the presence of ETEC in water was observed in fall and winter (100%). The LT-ETEC was only detected in summer 14.28% and in fall 40%. The positivity rate of ST-ETEC in water was highest in the winter, 100% and lowest in spring, 25%. The frequency of LT + ST-ETEC in water was 50% in the spring and monsoon and decreased to 14.28% in summer. The culturable ETEC were detected in water in spring (50%), summer (14.28%) and fall (20%) but not in the winter and monsoon.

Plankton exhibited a similar seasonal pattern of ETEC as water. All the plankton samples were positive for ETEC in the spring and winter (100%), while as in the water samples, the positivity rate decreased to 71.43% and 50%, in summer and monsoon respectively (Figure 2b). LT-ETEC was only detected in the spring, 25% and in summer 28.57%. The positivity rate of ST-ETEC in plankton followed similar pattern as in water, 40%–50% in spring, summer, and fall, however, was not detected in the winter. The occurrence of LT + ST-ETEC in plankton was highest in the winter (100%) and lowest in the spring (25%). The culturable ETEC was detected in plankton only in spring (25%).

The sediment samples showed a different seasonal pattern compared to water and plankton. All sediment samples tested positive for ETEC during spring, monsoon, and winter (100%), with a decrease in positivity rate to 80% during summer and fall (Figure 2c). Unlike water and plankton, sediment samples exhibited the highest positivity rate (100%) in the monsoon. LT-ETEC was only found in spring, with a detection rate of 33.33%. The positivity rate of ST-ETEC in sediment was highest in the summer, 80% followed by spring, 66.7% but was absent in winter. The occurrence of LT + ST-ETEC in sediment peaked in winter (100%) and was decreased to 20% in fall. Similar to water and plankton samples, culturable ETEC could not be detected in sediment in the monsoon and winter.

### 3.2 ETEC and environmental parameters

#### 3.2.1 pH

**FIGURE 3:**
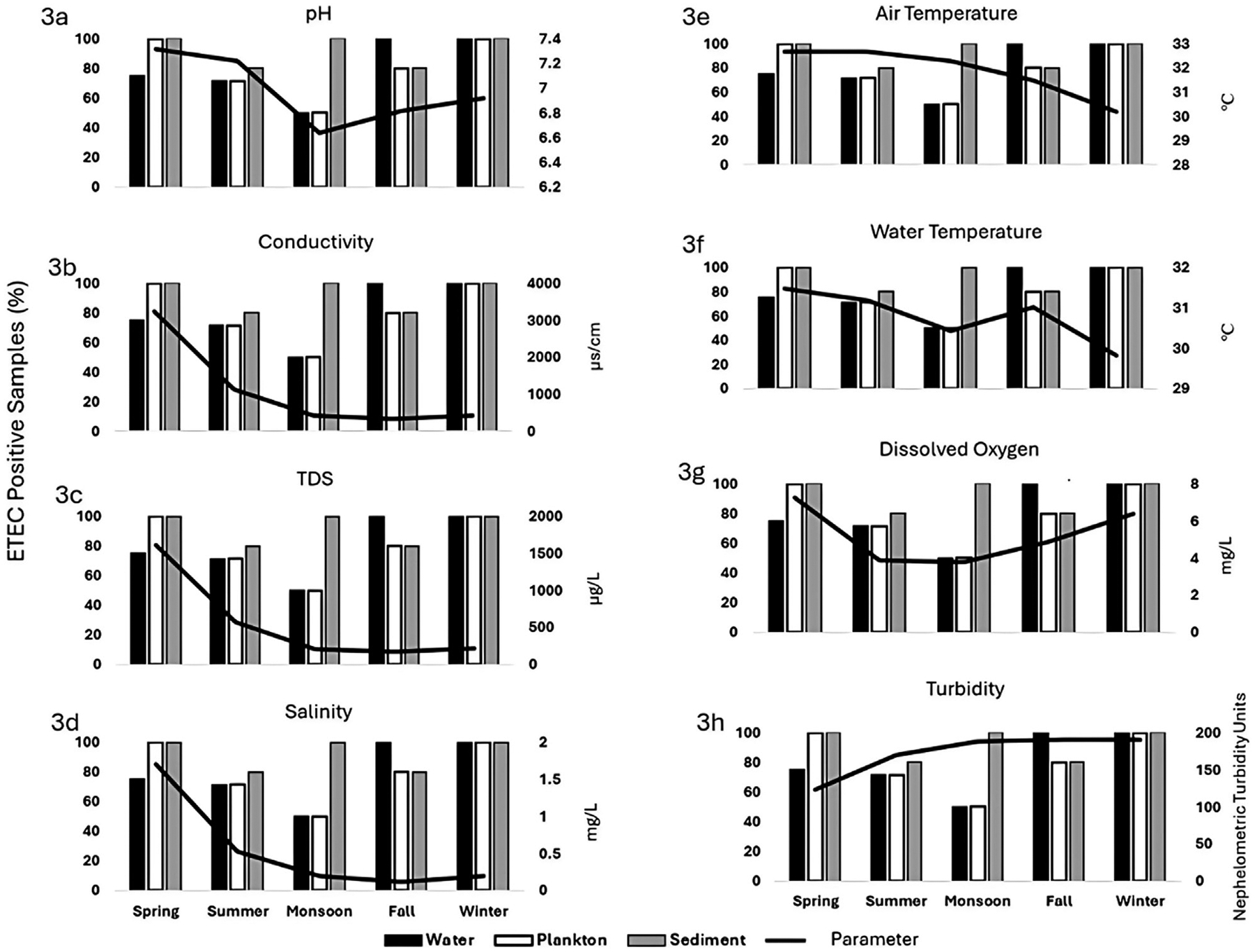
Relation between positivity rates of ETEC and physiochemical parameter in the aquatic environment in Mathbaria site 8: **(a)** pH, **(b)** conductivity, **(c)** total dissolved solid (TDS), **(d)** salinity, **(e)** air temperature, **(f)** water temperature **(g)** dissolved oxygen (DO), and (h) turbidity. X-axis represent seasons and Y-axis represent the number of ETEC positivity rate.

The average pH of water during the study period ranged from 6.64 to 7.315. In the spring, the average pH was 7.315, and as pH gradually decreased, the prevalence of ETEC also declined. In the fall, ETEC positivity increased as pH levels rose (Figure 3a). A similar trend was noted in the plankton samples that ETEC positivity increased as pH levels rose (OR = 3.364, p = 0.540, 95% CI: 0.060, 210.23) (Supplementary Tables S1–S5) while an opposite trend was found in the sediment samples where higher pH levels coincided with lower ETEC positivity (OR = 0.024, p = 0.304, 95% CI: 0.000, 9.478).

#### 3.2.2 TDS

The range of TDS in water during the study period was 214–1,614.5 μg/L. The highest TDS value was recorded in the spring (1,614.5 mg/L). The ETEC positivity in plankton somewhat mirrored the trend in water samples. As TDS decreased from spring to monsoon, ETEC positivity also decreased; however, in fall and winter, ETEC positivity increased despite lower TDS levels. No consistent trend was observed between TDS and ETEC positivity in sediment samples (Figure 3b).

#### 3.2.3 Conductivity

In spring, conductivity was highest (3,231.25 μs/cm), and ETEC positivity was 75%. As conductivity decreased to 420.5 μs/cm during the monsoon, ETEC positivity dropped to 50%. In fall and winter, ETEC positivity increased even as conductivity remained low. A similar trend was observed in plankton samples, with ETEC positivity decreasing as conductivity decreased from spring to monsoon, then increasing in fall and winter. No clear trend was observed between conductivity and ETEC positivity in sediment samples (Figure 3c).

#### 3.2.4 Salinity

The average range of salinity of water was 0.2 (mg/L) to 1.7 (mg/L). The highest ETEC positivity in water samples occurred in fall and winter when salinity was lowest (0.2–0.5 mg/L). In spring and summer, when salinity was higher (up to 1.7 mg/L), ETEC positivity was lower. Plankton samples followed the same trend while no consistent trend was observed between salinity and ETEC positivity in sediment samples (Figure 3d).

#### 3.2.5 Air temperature

The range of air temperature during the study period was 30.2°C–32.67°C. ETEC positivity in water samples increased as air temperature decreased from fall (32.67°C) to winter (30.2°C). No clear trend was observed between air temperature and ETEC positivity in plankton or sediment samples (Figure 3e).

#### 3.2.6 Water temperature

The water temperature varied from 29.83°C to 31.47°C In water samples, except in winter, ETEC positivity was higher when the water temperature was higher and decreased in monsoon as the temperature decreased. The ETEC positivity and water temperature followed the same positive trend in the plankton samples; however, the ETEC positive (31.27°C) plankton samples had a significantly higher mean water temperature than observed in ETEC negative (30.34°C) plankton samples (p = 0.005, 95% CI: −1.537, −0.328) (Supplementary Table S4). No clear trend was noted in the sediment samples (Figure 3f).

#### 3.2.7 Dissolved Oxygen

ETEC positivity rate and DO levels in water and plankton samples showed a positive trend. The ETEC positivity increased as DO levels rose. The lowest DO level (3.78 mg/L) during the monsoon coincided with the lowest ETEC positivity. In water samples, a near-significant trend was observed, with ETEC positivity increasing as DO levels rose (OR = 2.263, p = 0.263, 95%: 0.0857, 12.659). No clear trend was observed between DO and ETEC positivity in sediment samples (Figure 3g).

#### 3.2.8 Turbidity

ETEC positivity in water samples increased as turbidity rose during fall and winter. The highest ETEC positivity coincided with the highest turbidity levels in the seasons. Although the ETEC positivity rate was lower in the water and plankton samples in the monsoon even when the turbidity was higher, with near-significance in the plankton samples (p = 0.151, 95% CI: −17.16, 97.58) No clear trend was observed between turbidity and ETEC positivity in sediment samples (Figure 3h).

A comprehensive analysis of environmental parameters and ETEC presence was conducted across water, plankton, and sediment samples using logistic regression, Pearson’s correlation coefficient, and Welch’s t-test. Due to the small sample size by seasons, a significant relation was not always noted. The statistically significant and near-significant findings are included in the text. The near-significant trends suggest that the potential relationships may become statistically significant with a larger sample size. The detailed analysis is presented in the Supplementary Tables S1–S5.

## 4 Discussion

Our study shows a high prevalence of ETEC in the surface water of Bangladesh, both in the inland and coastal areas. For the first time, we showed planktons could harbor ETEC in high abundance, and culturable ETEC could also be isolated. The sediment in the water bodies was also highly contaminated with ETEC. The positivity rate of ETEC was observed to be influenced by the aquatic environmental parameters.

This study is the first to report the seasonal presence of ETEC in the water bodies in the coastal areas of Bangladesh. The aquatic ecology in these areas differs from that of inland areas, as the surface water in the coastal region can frequently mix with seawater of high salinity during floods, which are common in Bangladesh. Our study found that while ETEC was found all around the year in the water bodies, it was highest in the fall and winter, followed by spring, and lowest in the monsoon. Due to rain and an increase of water depth in the monsoon, the abundance of ETEC could be reduced in the water bodies, and due to the dry season and lower water depth, the abundance of ETEC increased in the winter. The positivity rate of ETEC was also high in the water, plankton, and sediments in the inland, northern hilly area, Chattak in Bangladesh. Interestingly, we found the seasonality and positivity rate of ETEC in plankton similar to that in water in Mathbaria. Although the positivity rates in the sediment samples were comparable, there is no distinct seasonal trend observed in these sediment samples.

A few studies were conducted in the central part of Bangladesh (in Dhaka) that showed 30% (by culture) (Begum et al., 2007) and 60% (by conventional PCR) (Akter et al., 2013) positivity rate of ETEC in the surface water, but these studies did not test planktons or sediments and did not find any seasonal variation. In our study, the positivity rates of the types of ETEC based on the presence of the toxin genes in the water and plankton did show seasonal variations. Interestingly, LT-ETEC was only identified in water during the summer and fall months. In plankton samples, LT-ETEC was present in both spring and summer, while in sediments, it appeared exclusively in spring, with a notably low abundance (14.28%–40%) across all types of samples. The highest prevalence was noted for ST-ETEC (25%–100%), followed by LT + ST-ETEC (20%–100%). Further investigations are needed to understand the influence of the environment on the survival and replication of the types of ETEC.

It’s noteworthy that ETEC diarrhea follows a seasonal pattern of two peaks every year, one in the pre-monsoon (March to May) and the second in post-monsoon (September to November) in the coastal area, Mathbaria (Chakraborty et al., 2024). This seasonal pattern of ETEC diarrhea matches that found in the aquatic bodies in this study, with very few to no ETEC positives during the monsoon. The positivity rates of the ETEC types in the aquatic samples in the same study period also matched with the ETEC types causing diarrhea cases that required hospital visits in Mathbaria (Chakraborty et al., 2024) with LT-ETEC, the lowest (5%), and ST-ETEC, the highest (65%) followed by LT + ST-ETEC (30%). These findings indicate a possible association between the abundance of aquatic ETEC and seasonal ETEC diarrhea outbreaks, which needs to be further studied.

We have used an extensive culture method to increase the sensitivity of isolation of culturable ETEC strains from the environmental samples. Interestingly, the culturable ETEC were isolated during pre-and post-Monsson but not during the Monsoon and winter, which matches the seasonality of ETEC diarrhea. Although the positivity rate of ETEC by qPCR was high in the winter in all three sample types, water, plankton, and sediment, but notably, no culturable ETEC were detected. It is possible that to survive the winter, ETEC could transform into viable but not culturable (VBNC) form and, with the onset of favorable environmental conditions in the spring, revert to culturable form. This survival mechanism of changing to VBNC during adverse ecological conditions was evidenced in *Vibrio cholerae* (Alam et al., 2007). Further studies are needed to understand the survival of ETEC in the environment in relation to seasonal changes.

This study showed that aquatic environmental parameters could drive the positivity rates of ETEC in the aquatic environment. Although due to the small sample size and data from only 1 year, statistical significance in correlations between the environmental parameters and ETEC was not found. However, environmental conditions such as neutral to low alkaline pH, elevated dissolved oxygen levels, and warmer water temperatures were associated with trends of increased ETEC prevalence.

This study has several strengths. This is a longitudinal investigation with serial sampling of water, plankton, and sediments conducted over almost a year. It is the first to show the abundance of ETEC and seasonal changes in the coastal aquatic environment, that ETEC could be detected and isolated from plankton, and that abundance varies by season. This work is the first attempt to determine the seasonal changes of ETEC in the aquatic environment and the role of the aquatic environmental parameters. Our study also has limitations. The sample size was small, and the data from only 1 year was collected. We were also unable to determine whether the ETEC strains isolated from environmental samples originated from human or animal sources. We tested the toxin genes but not the expression of these virulence factors and have not tested colonization factors and therefore if all of the identified ETEC strains will be able to cause diarrhea is uncertain. Although the data are not from the current years, the data are nevertheless valuable in showing new directions in the field to comprehend the impacts of the environment on the ETEC population.

## 5 Conclusion

This study is novel in several aspects and has established the importance of aquatic ecology in the survival and abundance of ETEC, which may influence the seasonal ETEC outbreaks in Bangladesh. This study has opened several questions that need further investigations. Will surveillance of water be useful to understand seasonal outbreak patterns of ETEC, similar to the use of wastewater to document the transmission of polio or COVID-19? Is the high abundance of ETEC in the water in winter without any viable ETEC isolation indicating the existence of VBNC ETEC? These questions could be answered in future studies with longitudinal serial sampling in shorter intervals of both clinical and environmental samples simultaneously over multiple years. The association of the environmental and clinical strains could be better understood by studying the lineages using whole genome sequencing of the ETEC isolates, which study is underway.

## Supplementary Material

Supplementary Tables S1-S5

The Supplementary Material for this article can be found online at: https://www.frontiersin.org/articles/10.3389/fenvs.2025.1593899/full#supplementary-material

## Figures and Tables

**TABLE 1:** Positivity rates of ETEC in the aquatic environmental samples determined by qPCR.

Location	Sample type	No. of samples	% Positive	LT-ETEC	ST-ETEC	LT + ST-ETEC
Mathbaria	Water	57	68.42% (39)	10.53% (6)	35.09% (20)	22.8% (13)
	Plankton	53	67.92% (36)	15.09% (8)	35.85% (19)	16.98% (9)
	Sediment	50	70% (35)	4% (2)	48% (24)	18% (9)
Chhatak	Water	9	77.78% (7)		22.22% (2)	55.56% (5)
	Plankton	9	77.78% (7)	22.22% (2)		55.56% (5)
	Sediment	9	55.55% (5)	11.11% (1)	11.11% (1)	33.33% (3)

## Data Availability

The original contributions presented in the study are included in the article/Supplementary Material, further inquiries can be directed to the corresponding author.
