# Supplementary material for: Occurrence of enterotoxigenic Escherichia coli in the aquatic environment and impact of climatic factors
Source: Front Environ Sci. Author manuscript; Available in PMC 2025 Dec 24. (PMC12724539; doi:10.3389/fenvs.2025.1593899)
Supplement: Supplementary Tables S1-S5 [file NIHMS2128269-supplement-Supplementary_Tables_S1-S5.docx]

Statistical analyses were performed to evaluate the relationship between ETEC presence and environmental parameters across water, plankton, and sediment samples. Univariate logistic regression was used to calculate odds ratios (OR) and 95% confidence intervals (CI) for each parameter. The odds ratio quantifies the likelihood of ETEC presence relative to its absence, with values greater than 1 indicated increased odds and values less than 1 indicating decreased odds. Pearson’s correlation coefficients were computed to assess the strength and direction of associations between environmental variables and ETEC presence. Welch’s t-test was employed to compare mean values of environmental parameters between ETEC-positive and ETEC-negative samples, accounting for unequal variances. All analyses were conducted using R (version 4.3.1, 2023-06-16), and significance was assessed at p < 0.05.

Table S1. Univariate, logistic regression results showing the odds of various samples containing ETEC by environmental parameter.

|  | Water Samples | | Plankton Samples | | Sediment Samples | |
| --- | --- | --- | --- | --- | --- | --- |
| Factors | Odds Ratio  (95% CI) | p-Value | Odds Ratio  (95% CI) | p-Value | Odds Ratio  (95% CI) | p-Value |
| pH | 0.485  (0.004, 25.252) | 0.730 | 3.364  (0.060, 210.228) | 0.540 | 0.024  (0.000, 9.478) | 0.304 |
| Total Dissolved Solids | 0.999  (0.998, 1.001) | 0.420 | 1.001  (0.999, 1.006) | 0.439 | 1.001  (0.998, 1.006) | 0.732 |
| Conductivity | 1.000  (0.999, 1.001) | 0.419 | 1.001  (1.000, 1.003) | 0.439 | 1.000  (0.999, 1.003) | 0.731 |
| Salinity | 0.564  (0.127, 2.727) | 0.420 | 4.561  (0.486, 695.278) | 0.406 | 1.657  (0.250, 373.581) | 0.718 |
| Average Water Temperature | 1.382  (0.448, 8.169) | 0.633 | 35.253  (1.502, 29831) | 0.130 | 1.726  (0.377, 45.021) | 0.613 |
| Air Temperature | 0.535  (0.141, 1.597) | 0.636 | 0.910  (0.296, 2.763) | 0.862 | 0.939  (0.220, 3.872) | 0.926 |
| Turbidity | 0.995  (0.972, 1.012) | 0.606 | 0.987  (0.957, 1.007) | 0.312 | 1.012  (0.994, 1.034) | 0.181 |
| Average Dissolved Oxygen Tension (DOT) | 2.263  (0.857, 12.659) | 0.263 | 1.581  (0.798, 6.887) | 0.461 | 1.021  (0.662, 2.861) | 0.943 |

Table S2. Pearson’s Correlation Coefficients comparing the association between environmental parameters and ETEC presence in various sample types.

|  | Water Samples | | Plankton Samples | | Sediment Samples | |
| --- | --- | --- | --- | --- | --- | --- |
| Factors | Correlation Coefficient  (95% CI) | p-Value | Correlation Coefficient  (95% CI) | p-Value | Correlation Coefficient  (95% CI) | p-Value |
| pH | -0.079  (-0.515, 0.389) | 0.746 | 0.142  (-0.334, 0.560) | 0.561 | -0.274  (-0.678, 0.257) | 0.305 |
| Total Dissolved Solids | -0.191  (-0.594, 0.288) | 0.433 | 0.193  (-0.286, 0.595) | 0.428 | 0.088  (-0.426, 0.559) | 0.745 |
| Conductivity | -0.192  (-0.594, 0.287) | 0.431 | 0.193  (-0.286, 0.595) | 0.428 | 0.088  (-0.426, 0.560) | 0.745 |
| Salinity | -0.192  (-0.594, 0.288) | 0.432 | 0.215  (-0.265, 0.610) | 0.377 | 0.094  (-0.422, 0.563) | 0.730 |
| Average Water Temperature | 0.111  (-0.362, 0.538) | 0.652 | 0.386  (-0.083, 0.715) | 0.102 | 0.131  (-0.390, 0.588) | 0.629 |
| Air Temperature | -0.252  (-0.634, 0.228) | 0.298 | -0.040  (-0.485, 0.422) | 0.871 | -0.023  (-0.513, 0.478) | 0.932 |
| Turbidity | -0.120  (-0.545, 0.353) | 0.624 | -0.242  (-0.627, 0.238) | 0.318 | 0.369  (-0.155, 0.731) | 0.160 |
| Average Dissolved Oxygen Tension (DOT) | 0.211  (-0.269, 0.607) | 0.386 | 0.172  (-0.306, 0.581) | 0.481 | 0.018  (-0.482, 0.509) | 0.948 |

Table S3. Comparing ETEC status and environmental parameters from water samples collected in Mathbaria, Bangladesh using Welch’s Two-Sample T-Test

| Environmental Parameter | Mean | | p-Value | 95% Confidence Interval |
| --- | --- | --- | --- | --- |
|  | ETEC Positive | ETEC Negative |  |  |
| pH | 7.048 | 7.102 | 0.772 | (-0.420, 0.529) |
| TDS | 565.2 | 862.2 | 0.570 | (-1071, 1665) |
| Conductivity | 1130 | 1727 | 0.570 | (-2148, 3341) |
| Salinity | 0.547 | 0.875 | 0.582 | (-1.246, 1.902) |
| Average Water Temperature | 31.13 | 30.86 | 0.527 | (-1.181, 0.646) |
| Air Temperature | 32.06 | 32.70 | 0.125 | (-0.205, 1.485) |
| Turbidity | 164.6 | 184.5 | 0.564 | (-58.74, 98.63) |
| Average DOT | 5.269 | 3.917 | 0.184 | (-3.431, 0.727) |

Table S4. Comparing ETEC status and environmental parameters from plankton samples collected in Mathbaria, Bangladesh using Welch’s Two-Sample T-Test

| Environmental Parameter | Mean | | p-Value | 95% Confidence Interval |
| --- | --- | --- | --- | --- |
|  | ETEC Positive | ETEC Negative |  |  |
| pH | 7.080 | 6.982 | 0.642 | (-0.637, 0.442) |
| TDS | 690.9 | 391.0 | 0.213 | (-793.3, 193.6) |
| Conductivity | 1382 | 781.5 | 0.213 | (-1587.9, 386.4) |
| Salinity | 0.693 | 0.325 | 0.159 | (-0.899, 0.162) |
| Average Water Temperature | **31.27** | **30.34** | **0.005** | **(-1.537, -0.328)** |
| Air Temperature | 32.17 | 32.28 | 0.871 | (-1.430, 1.634) |
| Turbidity | 160.3 | 200.5 | 0.151 | (-17.16, 97.58) |
| Average DOT | 5.217 | 4.112 | 0.236 | (-3.007, 0.797) |

Table S5. Comparing ETEC status and environmental parameters from sediment samples collected in Mathbaria, Bangladesh using Welch’s Two-Sample T-Test

| Environmental Parameter | Mean | | p-Value | 95% Confidence Interval |
| --- | --- | --- | --- | --- |
|  | ETEC Positive | ETEC Negative |  |  |
| pH | 6.981 | 7.210 | 0.493 | (-1.788, 2.247) |
| TDS | 614.5 | 432.5 | 0.579 | (-1085, 721.1) |
| Conductivity | 1229.6 | 864.5 | 0.578 | (-2167.8, 1437.5) |
| Salinity | 0.614 | 0.400 | 0.509 | (-1.011, 0.582) |
| Average Water Temperature | 31.20 | 30.80 | 0.202 | (-1.042, 0.242) |
| Air Temperature | 32.22 | 32.30 | 0.945 | (-7.424, 7.582) |
| Turbidity | 179.8 | 98.9 | 0.556 | (-1169.8, 1008.1) |
| Average DOT | 5.165 | 5.013 | 0.871 | (-2.148, 1.843) |
